# Efficacy of a Novel Metaldehyde Application Method to Control the Brown Garden Snail, *Cornu aspersum* (Helicidae), in South Africa

**DOI:** 10.3390/insects11070437

**Published:** 2020-07-13

**Authors:** Annika Pieterse, Antoinette Paula Malan, Jenna Louise Ross

**Affiliations:** 1 Department of Conservation Ecology and Entomology, Faculty of AgriSciences, Stellenbosch University, Private Bag X1, Matieland 7602, South Africa; annikapieterse@sun.ac.za (A.P.); apm@sun.ac.za (A.P.M.); 2 Institute of Biological and Environmental Sciences, University of Aberdeen, Aberdeen AB24 3UU, UK

**Keywords:** molluscicides, pests, apple orchards, IPM

## Abstract

Baitchain is a novel molluscicide system that consists of metaldehyde pellets arranged on a cord and is designed to be tied around the base of tree trunks and act as a physical and chemical control method for molluscs. In this study, Baitchain is tested in a South African apple orchard and compared with traditional metaldehyde pellets (Sluggit) applied to the soil surface to determine the efficacy of the products against the brown garden snail, *Cornu aspersum* (Helicidae). The products were applied at two different concentrations, including 15 g/kg (Baitchain 15 and Sluggit 15) and 40 g/kg (Baitchain 40 and Sluggit 40) metaldehyde, and the products were either applied on their own, or in combination. Both treatments at 40 g/kg metaldehyde caused significant snail mortality when applied either on their own or in combination. However, significant mortality was also achieved by Baitchain 15 when applied on its own as well as in combination with Sluggit 15. The increased efficacy achieved by using Baitchain, even at the lower concentration, indicates that this novel method offers a viable physical and chemical control option for molluscs and could be incorporated as part of an integrated pest management strategy in South Africa, as well as other parts of the world.

## 1. Introduction

In 2010, it was estimated that approximately 34 non-native terrestrial mollusc species were present in South Africa. Of the species, 28 are believed to have become established, with 13 being regarded as invasive species [1]. One such invader is the brown garden snail, *Cornu aspersum* (Müller, 1774) (Helicidae), which has been recorded from all nine provinces of South Africa [1]. During unfavourable seasonal conditions, the snail hibernates in the soil at depths of 10–20 mm, and when conditions improve, emerges for mating, laying eggs at a depth of 30–40 mm [2]. The snail then moves up into the trees, where it spends most of its time resting on the trunk, feeding at night or during moist periods. The snail feeds on developing foliar buds and young leaves in spring leading to stunted shoot growth and decreased yield of the crops. During the dry summer months, *C. aspersum* may aestivate by sealing the shell opening with an epiphragm [3]. In areas of extreme *C. aspersum* infestation, growers estimate that crop losses can reach up to 25% [4].

In South Africa, terrestrial mollusc pests are primarily controlled through the use of traditional chemical molluscicide pellets, usually containing metaldehyde, methiocarb or thiodicarb [5]. The pellets are composed mainly of wheat, bran or barley flour, which serves as an attractant, and which is combined with 2–8% of an active ingredient or toxicant [5]. The molluscs encounter the chemicals through feeding, or dermal contact, which causes the chemicals to act as stomach or contact poisons [6]. However, when overused, the chemicals can be toxic to nontarget organisms and can accumulate in the environment [7,8]. In addition, increased pressure has been exerted from regulatory bodies in regions such as Europe, in order to limit agricultural use of metaldehyde due to concerns relating to drinking water [8] and the impact on birds and small mammals [9]. Iron (Ferric) phosphate is another chemical or biorational control option that is effective at controlling slugs, offering less harmful impact to nontarget organisms [10,11,12]. However, the use of iron (Ferric) phosphate is less favourable than metaldehyde in South Africa due to cost restrictions [13].

The molluscicidal properties of metaldehyde were discovered in the 1930s in South Africa, at which point the chemical was being sold as fuel tablets [14]. Within four years, it became the most popular control method for use against terrestrial gastropod pests in countries such as the UK [14]. In 1996, it was estimated that metaldehyde was used on 55% of the crop areas where chemicals were being used against terrestrial gastropods [15].

In addition to chemical control, physical barriers can be used to control molluscs. A recent study reported that the use of a physical barrier, in combination with mineral oil and a snail-repellent paint (Sabzarang) containing copper and iron salts, was more effective at reducing the numbers of the citrus white snail, *Helicella candeharica* Pfeiffer (Panpulmonata: Helicidae) in citrus trees compared to surface broadcasting of metaldehyde or iron (Ferric) phosphate pellets applied to the soil [13].

The current study investigates the efficacy of a novel molluscicide application called Baitchain, which has been designed by the manufacturer to act as a physical and chemical control method. This new product, containing metaldehyde pellets arranged on a cord, was tied around the base of trees within an apple orchard and compared to traditional broadcasting of metaldehyde on the soil surface. The control potential of the different treatments was investigated by recording *C. aspersum* mortality over a period of 28 days.

## 2. Materials and Methods

### 2.1. Origin of the Chemicals

**Figure 1:**
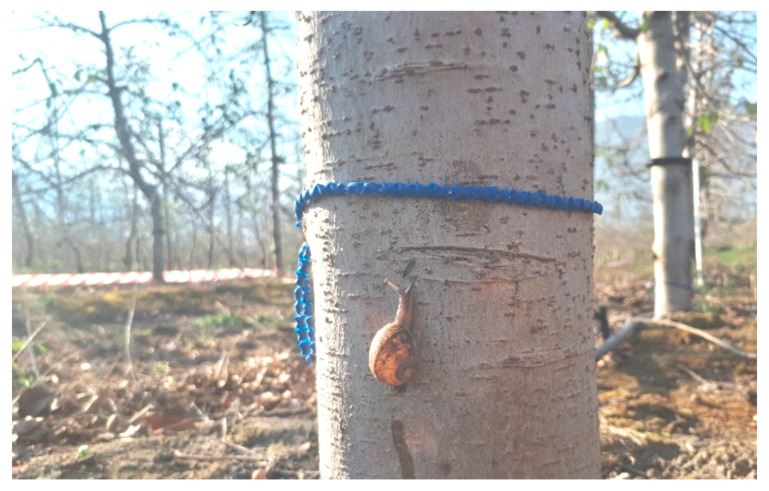
Baitchain applied to the trunk of an apple tree with *Cornu aspersum*.

The chemical products tested in the trial were obtained from the manufacturer, Orchard Suppliers CC, based in Worcester in the Western Cape province of South Africa. The efficacy of two different molluscicide products were tested including Baitchain (metaldehyde pellets on a cord; Figure 1) and Sluggit (a traditional metaldehyde pellet).

### 2.2. Test Area

The trial was conducted in August 2018 and repeated in August 2019, in an apple orchard (34°2′40.74″ S, 19°19′0.51″ E) outside Villiersdorp, in the Western Cape province of South Africa. Each of the 28 experimental plots measured 14 × 4 m in size. Each plot consisted of a row of six apple trees, planted 2 m apart, on a strip of bare soil, flanked by a 1-m wide strip of grass on each side. The plots were separated by a barrier of 2 m (Appendix A). The experimental layout consisted of four randomised blocks, each of which was separated into seven different treatment plots. Before treatments, the number of *C. aspersum* snails on each of the plots were counted by visually inspecting each plot for five minutes. The snails found in the trees were counted and placed on the soil surface inside the plot, so as to determine whether they were able to survive moving up the base of the tree and crossing the barrier treatment concerned. The location of the plots and the treatments used for each were the same in both seasons.

### 2.3. Treatments

Two concentrations were used for each product: Baitchain 15 and Sluggit 15, at 15 g/kg (1.5%) metaldehyde, and Baitchain 40 and Sluggit 40, at 40 g/kg (4.0%) metaldehyde. The treatments were: T1 = Baitchain 15; T2 = Sluggit 15; T3 = Baitchain 15 and Sluggit 15; T4 = Baitchain 40; T5 = Sluggit 40; T6 = Baitchain 40 and Sluggit 40; and T7 = control.

Sluggit pellets were applied at 8 kg/ha by broadcasting the pellets evenly throughout the plot, and Baitchain 15 and Baitchain 40 were applied at 4.79 kg/ha in plots, by tying the product around the base of the trunks, approximately 20 cm above the soil surface. Control plots consisted simply of a row of six apple trees on a strip of bare soil and flanked by a 1-m wide strip of grass on each side and were left untreated. The number of dead snails in the plots were counted by walking through each block for five minutes and physically removing dead specimens. Counts were done every 7 days, for 28 days, following application of treatments.

### 2.4. Statistical Analysis

The design of the experiment was essentially a split plot design, and a mixed model analysis of covariance was used for the data analysis. The blocks were entered into the model as a random effect. The fixed effects were the number of snails initially on the plot (covariate), treatment, and time. The dependent variable was the cumulative count of dead snails per plot. The interaction of treatment and time was the important effect investigated because it indicated different rates of cumulative increase over time. The normal probability plot was inspected to check for normality of the data and found to be in order. Fisher least significant difference (LSD) was used for post-hoc testing.

## 3. Results

**Figure 2:**
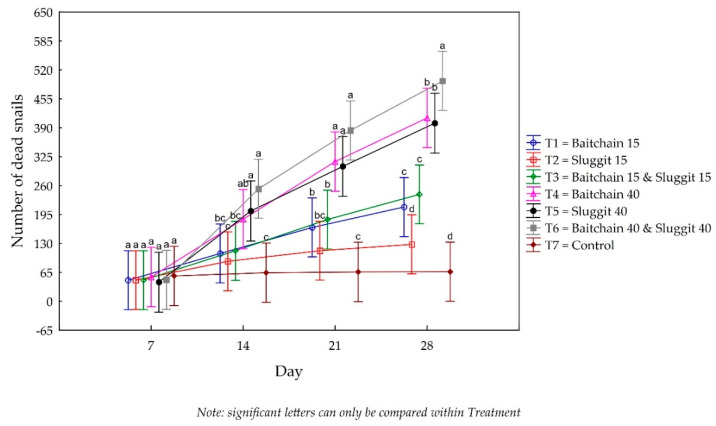
The number of dead snails counted (cumulative) every 7 days for 28 days, with six different concentrations and combinations of Baitchain 15, Sluggit 15, Baitchain 40 and Sluggit 40, as well as an untreated control. The same letters indicate no significant difference between the different treatments in the number of dead snails found. The vertical bars denote 0.95 confidence intervals.

The results of the trial show that Baitchain 15 (T1) had significantly more snail deaths than did the control by day 21. Sluggit 15 (T2) did not have significantly more snail deaths than did the control; however, when used in combination with Baitchain 15 (T1) in Baitchain 15 and Sluggit 15 (T3), significantly more snail deaths occurred than were found in the control on days 14 and 21 (F = 5.83; df = 18, 80; *P* < 0.01). All three treatments containing the higher dose (40 g/kg) of metaldehyde (T4, T5 and T6) caused significantly more snail deaths than were found to have occurred in the control on days 14, 21 and 28 (*F* = 5.83; *df* = 18, 80; *P* < 0.01). The combined treatment of Baitchain 40 and Sluggit 40 (T6) caused the highest snail mortality levels, significantly higher than was the number of dead snails caused by all the other treatments (*F* = 5.83; *df* = 18, 80; *P* < 0.01; Figure 2).

## 4. Discussion

Metaldehyde is usually applied in a compressed pellet form so as to slow down disintegration; however, it has also been applied by means of incorporating it into an edible matrix, which is then used to coat an inert granular core or in emulsified form as a spray [6]. In this study, a novel method of applying metaldehyde is tested and consists of metaldehyde pellets arranged on a cord. This system, called Baitchain, is designed to be tied around the base of tree trunks to act as a physical and chemical control method for molluscs. It combines compressed pellets with the quick-and-easy chain application system that can be applied by hand.

The results of this study showed that all the treatments at a metaldehyde concentration of 40 g/kg caused significant snail mortality. The 40 g/kg treatments caused significantly higher snail mortality compared with 15 g/kg treatments on most days during the trial, due to the much higher concentration of metaldehyde. However, Baitchain 15 applied on its own, and in combination with Sluggit 15, also caused significant snail mortality. The high number of dead snails found in the plots with 40 g/kg could be an indication of the amount present in the orchard and surrounding area. The higher concentration treatments simply killed more of the snails that entered the plots, whereas snails in plots with lower concentration treatments were able to survive or survive long enough to move outside of the plots. The efficacy of Baitchain, even at a relatively low concentration, indicates that this product offers a viable physical and chemical control option for molluscs in orchards. This novel approach could be investigated for use as part of a wider integrated pest management (IPM) strategy and incorporated into the Ross [9] mollusc IPM pyramid. However, further research is needed on a number of aspects of this product, including its ability to prevent crop damage, as well as its role in acting as a physical barrier. In the US, the University of California’s Statewide IPM Program (UCSIPMP) has defined guidelines for the integrated control of *C. aspersum* in citrus. The guidelines involve the application of a molluscicide early in the season, before the snails move into the trees, in combination with skirt-pruning of the trees, application of copper barriers to the trunks and the release of the predatory land snail, *Rumina decollata* (Linnaeus, 1758) as a biological control measure [16]. However, as *R. decollata* is not present in South Africa, it cannot be used under the terms of the Agricultural Pest Amendment Act, No. 18 of 1989, as it forbids the introduction of exotic species into South Africa [17]. In Australia, a combination of control methods are recommended, which include the monitoring of snail numbers, baiting, managing weeds and inter-rows, pruning tree skirts and using copper on tree trunks and foliage to deter snails [18].

Another biological control strategy is the use of the mollusc-parasitic nematode *Phasmarhabditis hermaphrodita* (Schneider, 1859) Andrássy, 1983 (Rhabditida: Rhabditidae) to control *C. aspersum*. The nematode is currently sold as a commercial biocontrol product by BASF and Dudutech, under the tradenames Nemaslug^®^ and Slugtech^®^, respectively [9,19]. However, the species has not, as yet, been found in South Africa, which prohibits its sale within the country. The potential of locally isolated mollusc-parasitic nematodes to control invasive molluscs in South Africa is currently being researched [20,21,22,23] and, should a candidate be found capable of infecting *C. aspersum*, could be used in combination with Baitchain products, as an elevated IPM strategy.

## 5. Conclusions

Bait pellets containing either 15 g/kg or 40 g/kg metaldehyde caused significant mortality of the brown garden snail, *C. aspersum* in apple orchard settings, when applied as a pellet to the soil surface, or as Baitchain wrapped around the base of trees or in combination. The efficacy of Baitchain, even at a lower metaldehyde concentration of 15 g/kg, means that it could possibly be used as part of a wider IPM strategy to control molluscs in orchards.

## Figures and Tables

**Figure A1:**
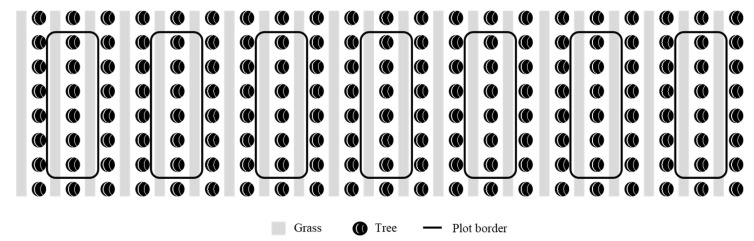
Layout of a single block in the orchard, showing seven plots, with one for each treatment.
